# Relationship quality in couples related to mental health of women and men during the COVID-19 pandemic and stay-at-home orders

**DOI:** 10.1192/j.eurpsy.2021.271

**Published:** 2021-08-13

**Authors:** L. Shaigerova, O. Vakhantseva, O. Almazova, R. Shilko, A. Dolgikh

**Affiliations:** 1 Faculty Of Psychology, Lomonosov Moscow State University, Moscow, Russian Federation; 2 Psychology, Lomonosov Moscow State University, Moscow, Russian Federation

**Keywords:** mental health, COVID-19, Gender differences, relationship quality

## Abstract

**Introduction:**

The data on gender differences in mental health make the investigation of the specific impact of the pandemic and of the stay-at-home orders on men and women relevant.

**Objectives:**

The study focuses on the quality of the relationship in couples and mental health in men and women during the COVID-19 pandemic.

**Methods:**

The study was conducted through an online survey a few weeks after the pandemic was declared and the stay-at-home order was introduced in Russia. 274 participants (50 men and 224 women) engaged in long-term relationships aged from 18 to 62 (M=34.2; SD=9.1) took part in the research. The instruments included the Warwick-Edinburgh Mental Well-Being Scale, the Perceived Relationship Quality Components, and the Depression Anxiety Stress Scales.

**Results:**

Women show a considerably higher level of stress (t=3.805; p<0.001), depression (t=3.76; p<0.001) and anxiety (t=2.959; p=0.003). The quality of relationship for women is significantly connected with mental wellbeing (r=0.423; p<0.001) and negatively correlated with the stress level (r= -0.60; p<0.001), depression (r= -0,381; p<0,001) and anxiety (r=-0,313; p<0,001). Meanwhile for men, the quality of the relationship is connected to mental wellbeing (r=0.280; p=0.049), opposed to stress levels (r= -0.316; p=0.025) and is neither connected to depression (r= -0.210; p=0.144) nor to anxiety (r= -0.126; p=0.383).

**Conclusions:**

During the pandemic, a favorable partnership has a positive effect on the mental health of both men and women. However, while the relationship quality affects all investigated indicators of mental health in women, in men the relationship quality is only connected to the level of mental wellbeing and stress. The reported study was funded by RFBR, project number 20-04-60174.

**Disclosure:**

No significant relationships.

